# The role of primary care in management of rare diseases in Ireland

**DOI:** 10.1007/s11845-019-02168-4

**Published:** 2020-01-13

**Authors:** Niall Byrne, Jacqueline Turner, Rita Marron, Deborah M. Lambert, Daniel N. Murphy, Grace O’Sullivan, Maureen Mason, Frank Broderick, Mary C. Burke, Sheila Casey, Marguerite Doyle, David Gibney, Fergus Mason, David Molony, Deirdre Ormond, Colm O’ Sé, Conor O’Shea, Eileen P. Treacy

**Affiliations:** 1 National Rare Disease Office, Eccles St, Dublin, D07 R2WY Ireland; 2 National Clinical Programme for Rare Diseases, HSE and RCPI, Dublin, Ireland; 3 Fairview Medical Centre, Fairview Strand, Dublin, Ireland; 4 Glasnevin Family Practice, Finglas Road, Glasnevin, Dublin, Ireland; 5 Kingscourt Surgery, Kingscourt, Co. Cavan, Ireland; 6 Aubrey Medical Centre, Shankill, Dublin, Ireland; 7 Ballymun Family Practice, Ballymun, Dublin, Ireland; 8 Johnstown Medical Centre, Cabinteely, Dublin, Ireland; 9 The Red House Family Practice, Mallow, Co., Cork, Ireland; 10 Windmill Medical Centre, Skerries, Co., Dublin, Ireland; 11 Ballyfermot Medical Centre, Dublin, Ireland; 12 Wheaton Hall Medical Practice, Co Louth, Drogheda, Ireland

**Keywords:** Clinical coding, General practitioners, Integrated delivery of healthcare, Patient care management, Rare diseases

## Abstract

**Background:**

‘Slaintecare’ aims to address complex patient care needs in an integrated fashion with an emphasis on patient-centred, patient-empowered community care.Currently there is a lack of knowledge of the impact of rare disease management in primary care and of the information tools required by general practitioners to deliver integrated care for rare disease patients.

**Aims:**

To complete a pilot survey to estimate the general practice clinical workload attributable to selected rare diseases and assess the use of relevant information sources.

**Methods:**

A retrospective cross-sectional survey was carried out of general practice consultations (2013–2017) for patients with 22 commonly recognised rare diseases.

**Results:**

Around 31 general practitioners from 10 Irish practices completed information on 171 patients with rare diseases over 3707 consultations. General practice-specific coding systems were inadequate for rare disease patient identification. Over 139 (81.3%) patients were adult, and 32 (18.7%) were children. Management of care was hospital and not primary care based in 63%. Those eligible for state-reimbursed care had a significantly higher median number of consultations (23 consultations, IQR = 13–37, or 5.8 consultations/year) than those who paid privately (10 consultations, IQR = 4–19, or 2.5 consultations/year) (*p* < 0.005).General practitioners had access to public information resources on rare diseases but few had knowledge of (35.5%), or had ever used (12.9%) Orphanet, the international rare disease information portal.

**Conclusions:**

Both specific rare disease-specific coding and use of the relevant rare disease information sources are lacking in general practice in Ireland.

## Background

In Europe, a disease is considered rare if it occurs in less than 5 per 10,000 persons. Rare diseases (RDs) are defined as life-threatening or chronically debilitating diseases with a high level of complexity. Most RDs are associated with chronic physical, intellectual or neurological disabilities. Collectively, they pose a significant economic burden [1–3]. The greater than 6100 known RDs are estimated to affect 3.5–5.9% of the population (excluding rare cancers and infectious diseases) equating to 263–446 million persons globally [4] and approximately 300,000 Irish people. 72% of RDs are genetic and 75% of RDs have paediatric onset [4]. However, the true prevalence of only a few rare disease in Ireland is unknown as there is currently no national RD registry and there are insufficient coding systems in place to capture RD prevalence [3]. Patients affected with RDs frequently describe a prolonged ‘diagnostic odyssey’ and significant difficulties in accessing coordinated care and treatment [1, 5–7].

The expansion of genomic testing will accelerate and expand the diagnosis of RDs [8]. With these developments and increased introduction of the ‘personalised care’ approach, there will be a concomitant need to develop clinical guidelines, clinical pathways, clinical decision support tools and patient information tools that are easily accessible at the point of care and that will empower patients in self-management [8, 9].

Adequate care coordination is particularly important for those affected by rare conditions. The UK 2011 National Coalition on Care coordination defined care coordination as ‘a person-centred, assessment-based, interdisciplinary approach to integrating health care and social support services in a cost-effective manner, in which an individual’s needs and preferences are assessed, a comprehensive care plan is developed and services managed and monitored by an evidence-based process’. Such coordination may be necessary, for example, when care is required over a long period of time; the level of care required increases; multiple services are needed; or a high frequency of emergency unplanned admissions occurs. For RDs, coordination across services is often essential and likely includes health, social, primary, secondary, tertiary and quaternary care, as well as voluntary sectors. Many patients will present with symptoms for the first time to their general practitioner (GP). Furthermore, most families of RD patients agree that their GP is in the position to coordinate the extensive range of services needed [10, 11].

Primary care can provide accessible and affordable care to RD patients and thereby reduces hospital admissions. GPs can guide RD patients through complicated hospital systems and coordinate care, potentially reducing both patient hardship and costs of care. Primary care teams offer psychological support to patients and their families and advocate access to expert healthcare in the community setting [11, 12]. In Ireland, state-reimbursed GP care is available to all people under 6 years or over 70 years or to people between 6 and 70 who qualify for a means-tested medical card; all others must privately pay for GP services.

The long-term plan for the UK NHS is to shift its emphasis from acute care hospitals to a new service model based on community care, population health and collaboration [13]. The Irish ‘Slaintecare’ plan also emphasises the enhanced integrated care of patients with chronic diseases in the community [14].

Primary care providers/general practitioners have a central role in health promotion and disease prevention. The National Clinical Programme for Rare Diseases has developed a model of care for RDs to improve access, value and quality of care for RD patients (https://www.lenus.ie/handle/10147/626904). This document highlights the unique role of the GP in coordinating care for RD patients. Knowledge of the underlying disorder and care pathway is crucial for the community treatment of individuals with a RD.

Orphanet is the globally used Europe-based website providing comprehensive information about orphan drugs and rare diseases, including information summaries, clinical practice guidelines and information about the location of expert resources, clinical research and trials, used by both physicians and patients. The website is managed by a consortium of academic establishments from 35 countries, led by Inserm (www.orpha.net) [15]. Ireland participates in the Orphanet consortium, and the Orphanet Ireland team based at the National Rare Diseases Office collects and curates information about national RD resources for the Orphanet database.

To date, there are no existing studies that explore the services provided by Irish general practice for people with RDs or the GPs’ use of RD resources. In this pilot survey, we explored the role of Primary Care/GPs in the Irish RD patient care journey.

## Methods

### Survey design

The survey was designed by National Rare Diseases Office (NRDO) staff to assess general practice involvement in the management of RD patient care in Ireland over a 4-year period (from June 2013 to June 2017). Documents produced for this survey included a list of 22 rare diseases with corresponding ICD-10 codes; a survey of the use of rare disease resources in primary care practices; and a questionnaire to be completed by the GP regarding each patient identified with one of the 22 rare diseases. The target of the survey was GPs registered with the Irish Medical Council or holding a place on the GP training scheme, in any size general practice, and in any county of Ireland.

About 22 RDs were chosen for survey on the basis of their relative high prevalence, chronic course and recognisability (Table 1). This survey did not include rare cancer diseases or rare infectious diseases that are outside the scope of the National Clinical Programme for rare diseases.

**Table 1:** Estimated European prevalence rates of the 22 selected Rare Diseases

Rare disease name	Estimated prevalence (per 100,000)
Congenital isolated hyperinsulinism Scleroderma (systemic sclerosis, localised sclerosis) Familial QT syndrome (Inc. Romano Ward) Primary systemic amyloidosis Retinitis pigmentosa Fragile X Neurofibromatosis 1 Marfan syndrome Sickle cell anaemia Sarcoidosis Haemophilia Huntington’s disease and rare neurodegenerative disease Phenylketonuria 22q11.2 Deletion syndrome (DiGeorge syndrome) Duchenne or Becker muscular dystrophy Prader-Willi syndrome Epidermolysis bullosa Mucopolysaccharidosis (type 2) Rare ataxias (including Friedreich’s) Tuberous sclerosis Osteogenesis imperfecta Motor neuron disease	2.0 *BP* 42.0 *P* 40.0 *BP* 30.0 *P* 26.7 *P* 32.5 *P* 21.3 *P* 15.0 *P* 22.0 *P* 12.5 *P* 7.7 *P* 2.7 *P* 10.0 *BP* 37.5 *BP* 4.78 *P/* 1.53 *P* 3.1 *BP* 2.2 BP * 10.0 *P* 2.0 P * 12.*0 P* 10.0 *P* 3.85 *P*

Estimates provided from the Orphanet Report Series

*P* = prevalence; *BP* = birth prevalence

*Estimates shown are for epidermolysis bullosa simplex and Friedreich’s Ataxia

Each participating GP was surveyed as to which resources (online/print) they would use to inform themselves when faced with a patient with a rare disease, as well as their knowledge and use of Orphanet.

Each GP was provided with questionnaires to complete for each patient they identified with one of the 22 surveyed rare diseases. This anonymised questionnaire included questions on (i) RD diagnosis; (ii) demographics (age group, gender, age of diagnosis, health insurance status); (iii) time demand (frequency of consultations per year, reasons for attending, type of consultations (face-to-face contact, phone calls and home visits)); (iv) the GP role(s) in care of the patient; (v) the GPs’ role in relation to the management of the patient; and (vi) the patient’s attendance at specialist appointments. Participants were asked to anonymize patient data with a random four-digit identifier prior to the questionnaire return to ensure patient confidentiality.

About 23 primary care centres of varying sizes (ranging from single practitioner to large practices) were invited via letter, email and telephone contact to participate. The practices were selected on a convenience sampling.

### Data collection and analysis

A designated staff member from each primary care/GP centre reviewed records and provided anonymised data to the research team.

Data were entered into a Microsoft Excel spreadsheet, and percentages, medians and interquartile ranges were calculated. Calculations were stratified by presence or absence of state-based care. Tests of significance were calculated using the two-sided Wilcoxon-Mann-Whitney test [16].

## Results

### Participating primary care centres

Ten primary care centres of the invited 23 centres participated in this survey. 31 GPs from these centres completed the surveys. 11 out of the 13 centres that did not take part indicated interest in participation but were unable due to clinic time pressures, and 2 did not indicate why they were unwilling to participate. The practices were located across four different counties, with staff ranging from one to approximately 7.5 whole-time GPs.

### Completion of questionnaire

Additional information was provided anecdotally by the GPs to the survey team during their visit. Data about rare disease diagnoses were not easily retrievable from their practice electronic records, and disease names and ICD-10 codes were of little assistance as search terms to identify RD patients. The practice IT systems used were reliant on ICPC-2 coding, International Coding on Primary Care, which is a patient problem-focused coding system and was not searchable by either the underlying rare disease diagnosis or ICD-10 code. ICD-9 was used in few of general practices. Retrieval of patients with the 22 diagnoses was frequently dependent on physician memory recall.

### Patient demographics

Data on 174 RD patients were returned from 10 primary care centres. About 171 patients had one of the 22 pre-identified common multi-systemic RDs (Table 2). Data were excluded for three patients whose diagnoses were not on the pre-identified RD list. The most common recorded RD was sarcoidosis (*n* = 63, 36.8%) followed by familial Long QT syndrome (*n* = 11, 6.4%), phenylketonuria (*n* = 10, 5.9%), scleroderma (n = 10, 5.9%), motor neuron diseases (*n* = 9, 5.3%) and rare neurodegenerative diseases (*n* = 8, 4.7%). About 54% (*n* = 92) of patients were male, and 46% (*n* = 79) were female. Most were adults (*n* = 141, 82% of the total) at the time of the survey. The age of diagnosis of the RD was adulthood in 69% (*n* = 118) of the patients. Around 57% (*n* = 97) of the RD patients had state-reimbursed medical care, and the remaining 43% were private fee-paying patients.

**Table 2:** Demographic features of the 171 Irish rare disease patients (2013–2017) surveyed from ten primary care practices

Variable	Rare disease patients *N* = 171	
	*N*	(%)
Gender		
Male	92	(54)
Female	79	(46)
Age (years) at time of survey		
≥ 18	141	(82)
< 18	30	(18)
Age (years) when diagnosed with RD (*N* = 171)		
≥ 18	118	(69)
< 18	53	(31)
Healthcare funding eligibility		
State-reimbursed care	97	(57)
Not state-reimbursed	74	(43)

*n* = number of patients.

Ages ranged from 3 to 85 years old

### Rare disease resources

The most commonly used resources by the GPs (*n* = 31), in order of decreasing popularity, were GP Notebook (17%), Google (15%) and specialist letters (12%) (see Table 3). Although 11 of the GPs surveyed (35.5%) were aware of Orphanet, only 4 GPs (12.9%) had used this in their practice.

**Table 3:** Preferred resources used by 31 surveyed Irish general practitioners to source rare disease information (2013–2017)

Resource(s) used	General practitioners *N* = 31	
	*N*	%
GP Notebook Google Specialist letter Patient.co.uk Medscape Royal College Hospital in Melbourne App Registered Charities Information from patient British Medical Journal Best Practice Journals	15 13 11 10 9 8 8 8 4 3	(17) (15) (12) (11) (10) (9) (9) (9) (4) (3)

### Patient attendance rates

The median attendance for all RD patients (*n* = 171) to GPs over the 4-year survey period was 16 (IQR = 8–33) or 4.0 visits per annum. Around 88.7% of these consultations were face-to-face, 11% were phone calls and 0.2% were home visits. When stratified by payment method, those with state-reimbursed care visited their GP significantly more frequently than those paying privately (median number of visits 23 (IQR = 13–37) compared to 10 (IQR = 4–19), respectively (*p* < 0.005)). There was no significant difference in type of consultation between the two payment groups.

### General practitioners’ role in patient care

Around 19% (*n* = 32) of patients had their RD diagnosed by their GP, and for a further 19% (n = 32), the GP played a major role in the diagnosis. Over 56% of patients (*n* = 96) obtained repeat prescriptions for their RD from their GP, 37% (*n* = 63) had complications of their RD managed by their GP and 16% (*n* = 28) were seen for routine RD management. Other reasons for attendance (*n* = 38, 22%) included taking bloods prior to specialist appointments, provision of psychological support and management of RD in pregnancy. Of the 147 patients actively managed at the time of survey completion, 63% (*n* = 93) of the patients had RD management only provided by specialists, 6% (n = 9) had the RD managed solely by their GP and 16% (*n* = 23) had their RD jointly managed by both a GP and a specialist. For the remaining 15% (*n* = 22), it was unclear who was managing the patient’s RD or whether the patient had been discharged from all services.

## Discussion

In this pilot survey, we have investigated the Irish general practitioners’ role in the care of a selected number of rare diseases and rare diseases information/educational tools used by GPs. We observed that RD patients who were eligible for state-reimbursed GP care (*n* = 97) had over twice the number of consultations (5.8 per year) compared to those who paid privately (*n* = 74) (2.5 per year). As the state-reimbursed category included means-tested and non-means-tested state reimbursement, it is difficult to draw any income-related conclusions from this data. However, as the cost burden has been alleviated for these patients, it could mean that they are more likely to attend their GP for guidance, management and support.

We found that 69% of RD patients surveyed were diagnosed in adulthood. This is a striking disparity considering 75% of RDs start in the paediatric period [4]. This may be accounted for by a bias of the selection of the diseases for the survey; 8 of the 22 diseases are exclusively of adult onset. Those conditions with adult onset have higher prevalence reported than the paediatric onset diseases surveyed (see Table 1). This pilot survey could not estimate the overall burden of RDs in general practice, as the survey included only 22 of the over 6000 RDs.

Our survey highlights the diverse roles of GPs in RD patient care, ranging from diagnostics to management of RD complications. For 38% of patients their GP was involved in their diagnosis either by making the diagnosis directly or playing a major role in the diagnostic work up of the patient. This compares to a recent Belgian study which showed that, for 121 RD patients, 36% of the time the GP established the diagnostic referral [17].

Our data suggest that most of the patients had their RD managed primarily by specialists, while some GPs were unaware of whom was the primary physician managing the patient. These data correspond with the Belgian experience which reported that GPs chose to refer RD patients to specialists rather than directly consulting the specialists to help reach a diagnosis [18]. Patients who are managed entirely by specialists alone may miss out on support and allied health services that are available at a local level.

The GPs in this survey did not generally use Orphanet. Only 35.5% of GPs surveyed were aware of Orphanet and an even lower percentage of users surveyed (12.9%) use this resource, comparable to the Belgian study, which showed that Orphanet was used by only 10 of the 64 (16%) GP practices with RD patients [18]. Utilisation of Orphanet would be helpful to GPs as it contains centralised, curated and current information about Irish rare disease resources. Other popular resources among GPs may provide a general overview of RDs, but they offer little information beyond this, such as details on pathophysiology, locations of centres of expertise, support groups, current research and clinical trials.

Both our survey and the Belgian study point to the lack of a specific RD coding system used in general practice. GPs have difficulty identifying the RD patients in their practices through use of the electronic system, often relying on memory. The use of a specific RD coding system, such as ORPHAcodes, as part of the patient’s electronic health record would allow for the burden of RDs in general practice to be measured [3]. Such an approach is being adopted elsewhere: in England, the move to a single terminology, SNOMED CT, was implemented across primary care and began to be deployed to GP in a phased approach from April 2018 [19, 20]. Currently in Ireland, the implementation of the SNOMED CT has been agreed for retro-fit to GP software, which will facilitate more widespread and accurate recording of rare diseases. However, the EU Commission 2009 recommendations regarding coding, recently reiterated by the EC Steering Group on Health Promotion, Disease Prevention and Management of Non-Communicable Diseases (SGPP) Committee, has recommended adding ORPHAcodes to national health information systems [21].

Access to a shared electronic health record could improve communication and enhance patient care by clarifying which health care providers are actively managing the patient and allowing co-ordination of care between primary and specialist care. Ireland is currently lacking a shared electronic health record system linking hospitals and primary care centres. This initiative is planned for the proposed Slaintecare health programme to parallel the Northern Ireland Electronic Care Record (NIECR) [22]. A shared record would allow RD codes to be assigned to the patient at diagnosis and to remain associated with the patient record so repeat coding for chronic conditions would not be required.

### Survey limitations

Recruitment of centres for this survey proved to be a challenging task, reflected in the poor response rate. The resulting major limitation of this survey is the small sample of patients due to the small number of participating GPs. The reliance of physician memory recall in many instances to identify subjects with one of 22 rare diseases may also have led to under-ascertainment of patients in this survey. Without Irish reference data on the prevalence of individual rare diseases, it is not possible to test whether the expected number of cases of each diagnosis was obtained in this survey or assess the degree of under-ascertainment. In the absence of centralised electronic health records, it is not possible to track RD patients that may have left the participating GP practices or who may have died over the survey period.

As a pilot survey, it does however shed some light into the RD patient journey involving primary health care. A larger survey would be necessary to provide an adequate sample size to generate sufficient data for rigorous statistical analysis. However, the feedback from the non-participating practices indicated that busy competing clinical pressures limited the ability to retrieve and collate data, greatly compounded by inadequate primary care IT and coding systems for RDs.

## Conclusions

In conclusion, this survey indicates that management for RD patients in Ireland is more likely to be managed by specialist consultants than in primary care.

Further evaluation of the two-way communication between primary care and specialist health care services is warranted given that there is on occasion uncertainty around which physician leads the management in the care of some patients with rare diseases. Introduction of RD coding nomenclature and shared electronic health records would permit RD patients to be identified within primary care systems to enable future RD healthcare planning and facilitate epidemiological research. It is hoped in the future that enhanced education in primary care and use of the existent national RD educational resources (www.rarediseases.ie) and Orphanet (www.orpha.net) will assist with improving the coordination and care of RD patients in the community settings.
